# Primary Ovarian Leiomyosarcoma: A Case Report and Review of the Literature

**DOI:** 10.7759/cureus.78005

**Published:** 2025-01-26

**Authors:** Adil Elghanmi, Fadila Kouhen, Leila Abdallaoui Maane, Karima Fichtali, Bouchra Ghazi

**Affiliations:** 1 Immunopathology-Immunotherapy-Immunomonitoring Laboratory, Faculty of Medicine, Mohammed VI University of Health and Sciences (UM6SS), Casablanca, MAR; 2 Department of Gynecology and Obstetrics, Mohammed VI International University Hospital, Bouskoura, MAR; 3 Laboratory of Neuro-Oncology, Oncogenetics and Personalized Medicine, Faculty of Medicine, Mohammed VI University of Health and Sciences (UM6SS), Casablanca, MAR; 4 Department of Radiotherapy, Cheikh Khalifa International University Hospital, Bouskoura, MAR

**Keywords:** ct (computed tomography) imaging, ovarian tumor, pelvic mass, primary leiomyosarcoma, surgery

## Abstract

Leiomyosarcoma, a rare malignant mesenchymal tumor originating from smooth muscle, comprises a small proportion of all ovarian malignancies and presents as an aggressive ovarian tumor.

This case report presents a 56-year-old postmenopausal woman diagnosed with ovarian leiomyosarcoma (OLMS). The patient presented with chronic pelvic pain, abdominal distension, and metrorrhagia. Physical examination revealed a 12 cm pelvic mass in the right adnexal region. The surgical intervention included total hysterectomy with bilateral adnexectomy, bilateral pelvic and para-aortic lymphadenectomy, and total omentectomy, confirming leiomyosarcoma via histopathological analysis.

The literature review underscores the absence of established pathogenesis for primary ovarian leiomyosarcoma (POLMS), highlighting diagnostic challenges due to the lack of specific tumor markers and ambiguous imaging results. Despite aggressive management, POLMS exhibits a poor prognosis for advanced tumors. Given the rarity of reported cases and the limited understanding of optimal management strategies, this report seeks to improve knowledge of the diagnostic challenges and therapeutic approaches for OLMS, emphasizing its atypical presentation and treatment considerations.

## Introduction

Ovarian leiomyosarcoma (OLMS) is a rare and aggressive malignant tumor arising from smooth muscle, representing less than 0.1% of all ovarian malignancies [1,2]. Due to its mesenchymal origin, OLMS is distinct from the more common epithelial ovarian cancers and poses unique diagnostic and therapeutic challenges. This rare entity predominantly occurs in postmenopausal women, though cases in younger individuals have been reported [1,3]. Its clinical presentation often involves the rapid development of a unilateral adnexal mass, accompanied by nonspecific symptoms such as lower abdominal pain, discomfort, or vaginal bleeding [1,2]. Unlike many other ovarian malignancies, serum levels of cancer antigen-125 (CA-125), a commonly utilized tumor marker, often remain within normal limits or show only mild elevation, adding to the diagnostic complexity [4,5].

The primary treatment modality for OLMS is radical surgical intervention, which typically includes abdominal hysterectomy, bilateral salpingo-oophorectomy, and omentectomy [6]. However, the definitive diagnosis of OLMS relies on histopathological evaluation, supplemented by immunohistochemical analysis to confirm the tumor's mesenchymal origin and rule out other differential diagnoses. Despite advances in surgical techniques and multimodal treatment approaches, the prognosis for OLMS remains poor due to its aggressive nature, high metastatic potential, and resistance to conventional chemotherapy and radiotherapy [1,7-9].

This article presents a case of postmenopausal OLMS, shedding light on its clinical features, diagnostic hurdles, and therapeutic challenges. By highlighting the intricacies of managing this rare malignancy, we aim to contribute to the limited body of literature and provide insights into improving early detection and outcomes for affected patients.

## Case presentation

We present the case of a 56-year-old Moroccan woman, postmenopausal for five years, who reported persistent chronic pelvic pain lasting for one year. Her symptoms were later accompanied by metrorrhagia, unintentional weight loss of approximately 4 kg over six months, and a noticeable decline in her overall health status. Her medical history included a myomectomy at the age of 31 for uterine fibroids.

On clinical evaluation, the patient had a body mass index of 31 and stable vital signs. Abdominal examination revealed marked abdominopelvic distension, with a firm, non-mobile mass approximately 12 cm in diameter, palpable in the right adnexal region and extending toward the umbilicus. Gynecological examination showed normal external genitalia and a soft cervix, without any abnormal vaginal discharge or palpable cervical anomalies.

**Figure 1:**
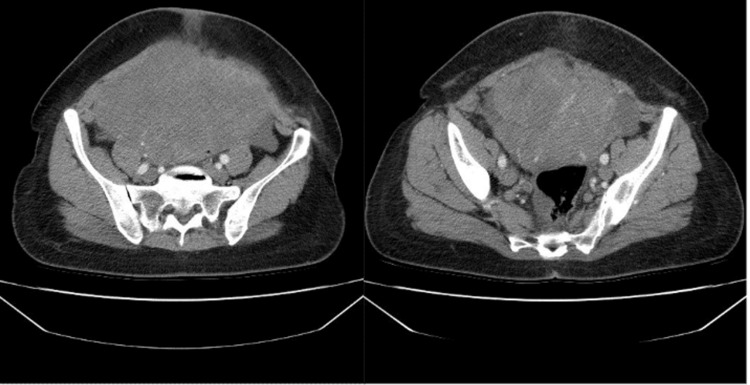
CT scan demonstrating a 12 cm pelvic mass in the right adnexal region with irregular borders, central necrosis, and displacement of adjacent digestive structures. CT, computed tomography

Laboratory investigations showed mild elevation of CA-125 at 51 U/mL, with all other tumor markers, including alpha-fetoprotein (AFP) and human chorionic gonadotropin (hCG), within normal limits. Pelvic ultrasound revealed a complex, cystic-solid mass measuring approximately 12 cm, originating from the right adnexa, with internal vascularity noted on Doppler imaging. A subsequent computed tomography (CT) scan confirmed the presence of a solid-cystic mass with irregular borders and partial necrosis, suggestive of malignancy (Figure 1). There was no evidence of lymphadenopathy, ascites, or distant metastases.

**Figure 2:**
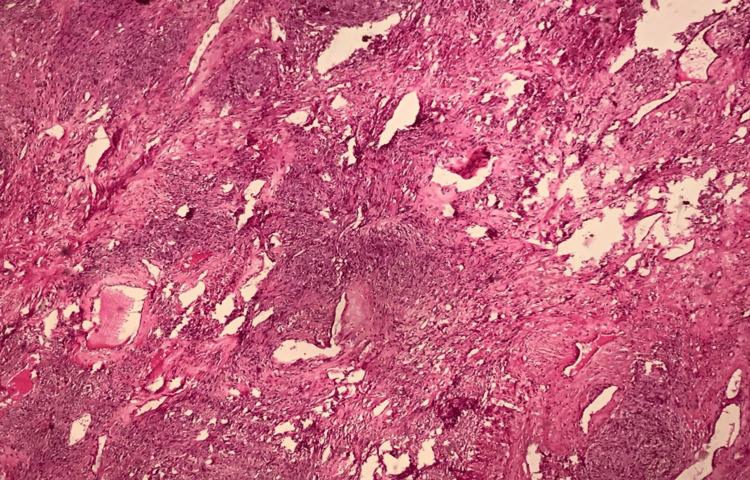
Mesenchymal tumor proliferation of spindle cells arranged in long, intersecting bundles; hematoxylin and eosin staining; and optical microscopy.

**Figure 3:**
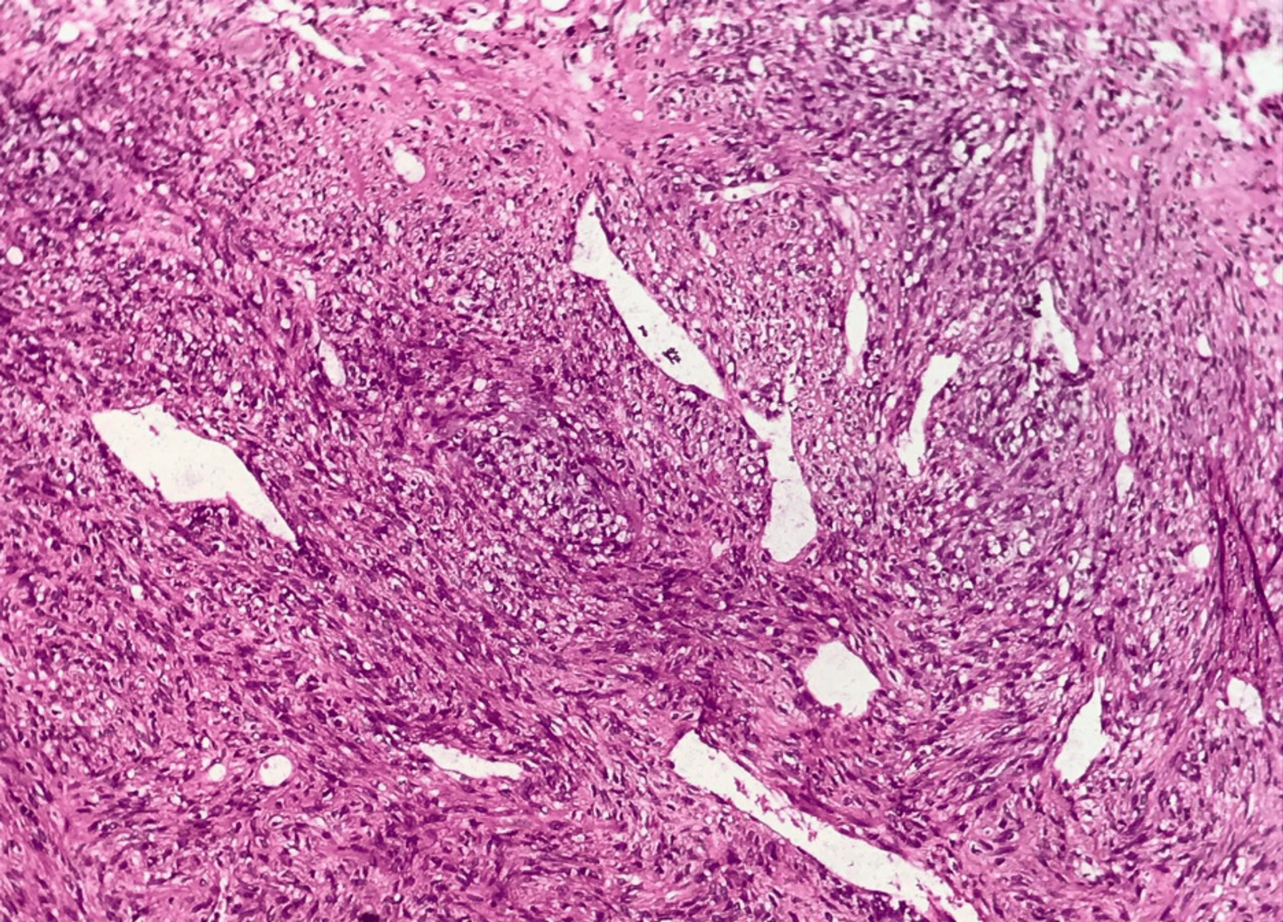
Tumoral stroma with highly developed vascularization of the hemangiopericytic type.

**Figure 4:**
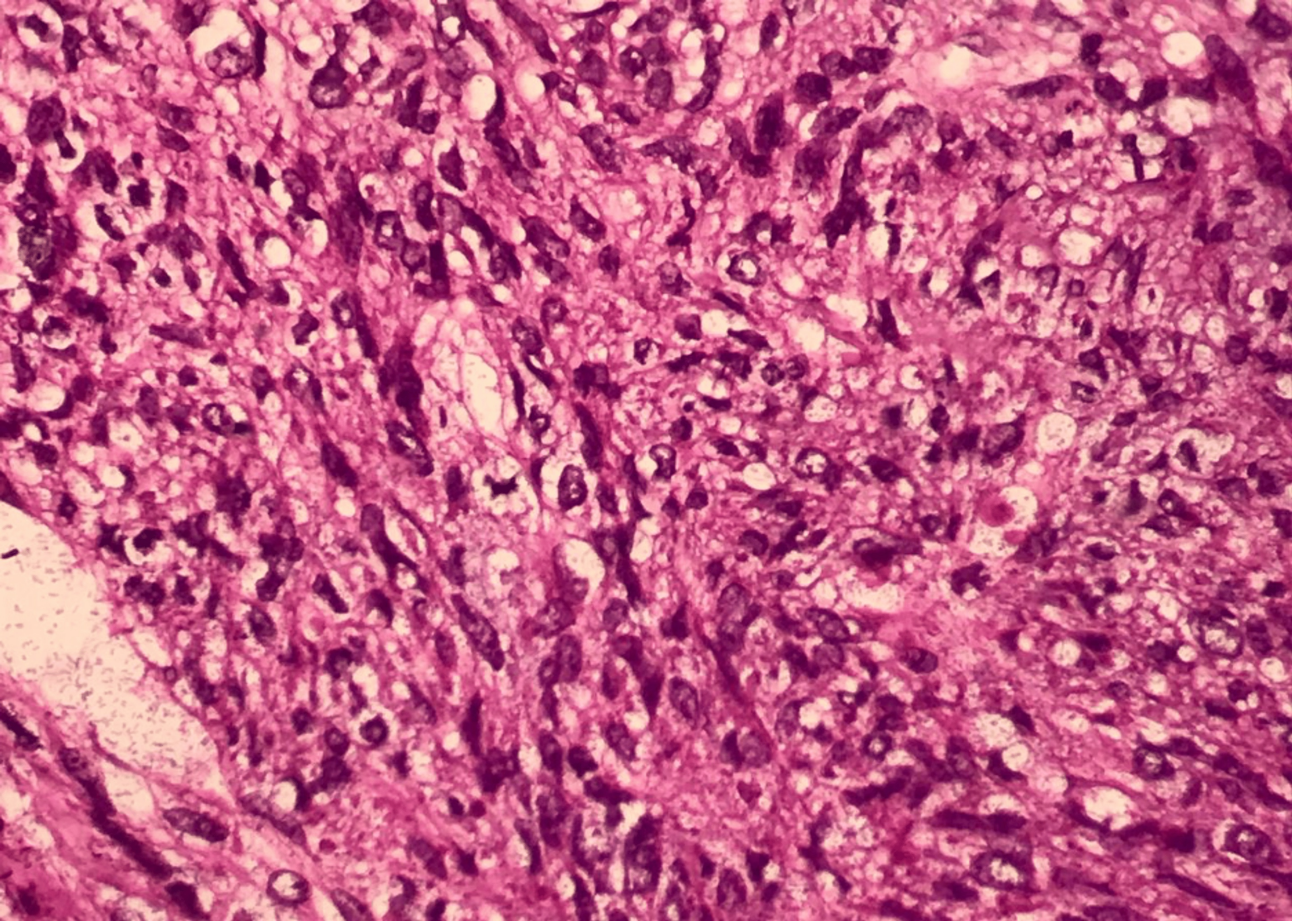
Tumor cells contain hyperchromatic nuclei with severe anisokaryosis and abnormal mitosis; hematoxylin and eosin staining; optical microscopy at ×40 magnification.

**Figure 5:**
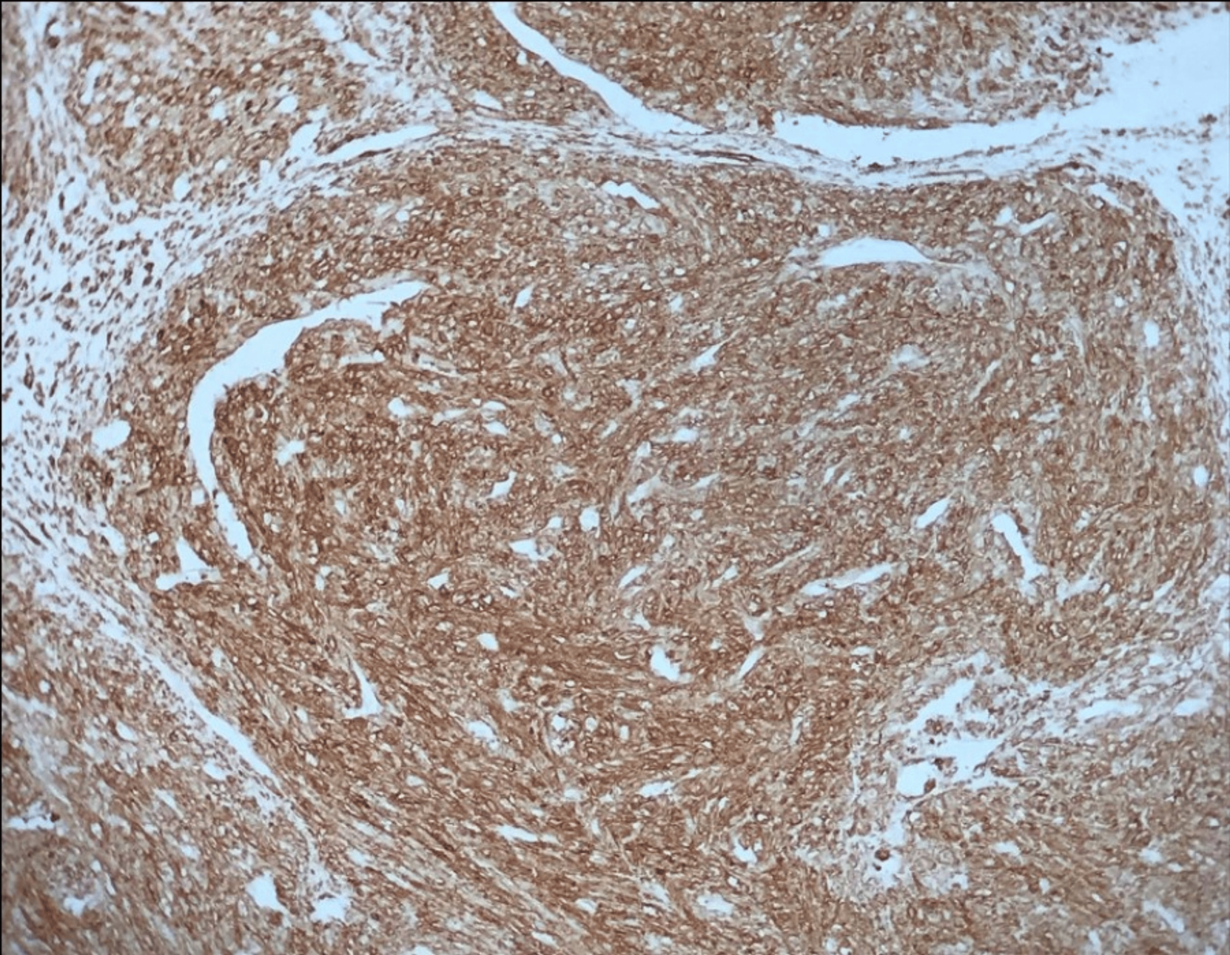
Immunohistochemical study showing strong expression of smooth muscle actin antibody by the tumor cells.

Given the tumor's characteristics, an exploratory laparotomy was performed, revealing a 12-cm right ovarian mass with dense adhesions to the uterus and low-grade ascites, which cytological analysis later confirmed as negative for malignant cells. A total hysterectomy, bilateral salpingo-oophorectomy, pelvic and para-aortic lymphadenectomy, and total omentectomy were conducted. Postoperative pathology confirmed a diagnosis of OLMS, characterized by spindle-shaped malignant mesenchymal cells, high mitotic activity, nuclear pleomorphism, and areas of necrosis (Figures 2-4). Immunohistochemical analysis demonstrated positivity for desmin, smooth muscle actin (SMA), and h-caldesmon, supporting smooth muscle origin (Figure 5).

The patient’s postoperative recovery was uneventful. She completed six cycles of adjuvant chemotherapy with doxorubicin and ifosfamide. Follow-up pulmonary and abdominal imaging over 12 months showed no evidence of recurrence or metastasis, and the patient remained asymptomatic and in stable condition. This case highlights the rarity and diagnostic challenges of OLMS, emphasizing the importance of comprehensive surgical management and follow-up in ensuring favorable outcomes.

## Discussion

Leiomyosarcomas originating from the ovary are exceptionally rare, representing less than 0.1% of all ovarian malignancies, with their histogenesis remaining unclear [1]. These tumors are thought to arise from the smooth muscle component of the ovary or potentially from the walls of ovarian blood vessels, but definitive origins are yet to be fully elucidated [2]. Their rarity poses significant challenges in understanding their clinical behavior, optimal management, and prognosis, leaving clinicians to rely largely on extrapolated data from uterine leiomyosarcomas and other soft tissue sarcomas [1,3,9,10].

Our patient, a 56-year-old postmenopausal woman, fits the demographic profile commonly associated with OLMS. While predominantly seen in postmenopausal women, these tumors have also been reported in younger individuals, including adolescents, demonstrating a wide age distribution [1-3,8].

The clinical presentation of POLMS often includes nonspecific symptoms such as abdominal pain, distension, and palpable masses. Additional symptoms, such as loss of appetite, urinary difficulties, and abnormal uterine bleeding, though less common, have been documented [1,2,6]. In our case, chronic pelvic pain and recent metrorrhagia were the key presenting features. Such nonspecific symptoms often contribute to delayed diagnosis, as they mimic those of more common ovarian pathologies.

Preoperative diagnosis remains a significant hurdle due to the tumor's ambiguous imaging features. Ultrasound, while commonly used, often fails to definitively distinguish between benign and malignant ovarian masses, as both may exhibit solid and cystic components [4-6]. Magnetic resonance imaging (MRI) with contrast, although more accurate in delineating malignancy, was not employed in this case. Instead, a contrast-enhanced CT scan suggested malignancy, revealing a solid-cystic adnexal mass with irregular borders. Tumor markers, such as CA-125, are frequently normal or only mildly elevated, as seen in our patient. However, the lack of specificity of CA-125 necessitates its use in combination with other diagnostic tools to enhance accuracy [4-6].

Histopathological evaluation remains the cornerstone of diagnosis for OLMS. As definitive preoperative criteria are unavailable, diagnostic confirmation relies on histological and immunohistochemical analysis, often adapted from uterine leiomyosarcoma criteria [3]. These tumors typically exhibit spindle cells, nuclear pleomorphism, a high mitotic rate, and areas of necrosis. Immunohistochemistry further aids in diagnosis by confirming smooth muscle origin through markers such as desmin, SMA, h-caldesmon, vimentin, and global muscle actin [1,6,8,11]. The absence of these markers can prompt consideration of alternative diagnoses, such as undifferentiated carcinoma or mixed Müllerian tumors, underscoring the importance of comprehensive analysis [2].

Surgical resection remains the primary treatment modality and the cornerstone of management for POLMS. Complete tumor removal, typically involving total abdominal hysterectomy, bilateral salpingo-oophorectomy, and omentectomy, is critical to achieving optimal outcomes. In our patient, this extensive surgical approach was undertaken successfully, and pathology confirmed no residual disease or lymph node involvement [6,8,12].

Although surgery addresses the primary tumor, POLMS is prone to local recurrence and distant metastases, most commonly to the lungs and bones [1,6]. Adjuvant chemotherapy is often employed postoperatively, particularly in high-risk cases or where metastases are suspected. However, leiomyosarcomas are known for their low chemosensitivity and radioresistance, contributing to high recurrence rates and poor overall survival [1,8,11,13]. Standard chemotherapy regimens are not well established, but combinations such as doxorubicin and ifosfamide have shown modest efficacy. Our patient received six cycles of adjuvant chemotherapy and remained disease-free at her 12-month follow-up.

Emerging evidence suggests that lymphadenectomy, when combined with chemotherapy, may improve survival outcomes in patients with POLMS. However, due to the tumor's rarity, data remain sparse, and no consensus guidelines exist regarding optimal treatment protocols. Further research into targeted therapies, advanced imaging techniques, and molecular profiling is urgently needed to improve the understanding and management of this aggressive malignancy. Despite these efforts, the prognosis for OLMS remains poor, with overall survival rates limited by high recurrence and metastatic potential [8,9]. This underscores the importance of long-term surveillance and multidisciplinary care for affected patients.

## Conclusions

POLMS is an exceptionally rare and aggressive malignancy with a poor prognosis. Diagnosis is often challenging due to nonspecific symptoms and imaging findings, requiring histopathological and immunohistochemical confirmation. Surgical resection remains the primary treatment, with adjuvant chemotherapy used despite limited efficacy. This case underscores the importance of a multidisciplinary approach and highlights the need for further research to improve outcomes for this rare tumor.
